# Selenium-Enriched Mushroom Powder Enhances Intestinal Health and Growth Performance in the Absence of Zinc Oxide in Post-Weaned Pig Diets

**DOI:** 10.3390/ani12121503

**Published:** 2022-06-09

**Authors:** Eadaoin Conway, Torres Sweeney, Alison Dowley, Stafford Vigors, Marion Ryan, Supriya Yadav, Jude Wilson, John V. O’Doherty

**Affiliations:** 1 School of Agriculture and Food Science, University College Dublin, Belfield, D04 V1W8 Dublin, Ireland; eadaoin.conway@ucdconnect.ie (E.C.); alison.dowley@ucdconnect.ie (A.D.); stafford.vigors@ucd.ie (S.V.); 2 School of Veterinary Medicine, University College Dublin, Belfield, D04 V1W8 Dublin, Ireland; torres.sweeney@ucd.ie (T.S.); marion.ryan@ucd.ie (M.R.); 3 MBio, Monaghan Mushroom Group, Tyholland, H18 FW95 Monaghan, Ireland; supriya.yadav@mbio.ie (S.Y.); jude.wilson@mbio.ie (J.W.)

**Keywords:** pigs, post-weaning, selenium, microbiota, β-glucan, zinc oxide, *Agaricus bisporus*

## Abstract

**Simple Summary:**

The imminent ban on zinc oxide in pig diets within the European Union is a major challenge facing the swine industry. Commercial weaning is associated with abrupt dietary, environmental and social changes resulting in stress, reduced feed intake and gut developmental issues in the post-weaned pig. Mushrooms are rich in natural bioactives and have long been regarded as a health-promoting food due to their immunomodulatory and antioxidant effects and their ability to modulate the gut microbiota. Mushrooms can become abundant in organic selenium when grown under certain conditions. The present study aimed to determine the optimum Se level, using Se-enriched mushrooms and selenite, in weaned pig diets to enhance intestinal health, performance and antioxidant capacity. Our study demonstrated that 0.3 ppm selenium inclusion, using selenium-enriched mushroom powder, led to positive effects on faecal scores and had similar pig performance compared to zinc oxide during the first 21 days post-weaning. The selenium inclusion level of 0.6 ppm, using selenium-enriched mushroom powder and selenite, enhanced pig performance and aspects of gastrointestinal health during days 21 and 39 post-weaning.

**Abstract:**

This study was conducted to examine the effects of varying selenium (Se) inclusion levels, in the form of Se-enriched mushroom powder (SeMP) and selenite, on post-weaning growth performance (Period 1; day 1–21), intestinal health and antioxidant capacity (Period 2; day 21–39). Weaned pigs were blocked according to live weight, sex and litter of origin and randomly assigned to the following experimental groups: basal (basal + selenite (0.3 ppm Se)); ZnO (basal + ZnO + selenite (0.3 ppm Se)); 0.15 SeMP (basal + SeMP (0.15 ppm Se)); 0.3 SeMP (basal + SeMP (0.3 ppm Se)) and 0.6 SeMP/Sel (basal + SeMP (0.3 ppm Se) + selenite (Sel) (0.3 ppm Se)) with eight replicates/experimental group. After 21 days, the ZnO experimental group was removed from the experiment and the remaining pigs continued on their respective diet until day 39 post-weaning (Period 2). In Period 1, 0.15 SeMP supplementation reduced (*p* < 0.05) average daily gain (ADG), average daily feed intake (ADFI) and day 21 body weight, and increased (*p* < 0.05) faecal scores compared to the ZnO group. Supplementation with 0.3 SeMP and 0.6 SeMP/Sel during Period 1 resulted in similar (*p* > 0.05) ADG, ADFI, gain-to-feed ratio (G:F) and body weight compared to the ZnO group. However, 0.6 SeMP/Sel supplementation increased (*p* < 0.05) faecal scores compared to the ZnO group. In Period 2, 0.6 SeMP/Sel increased (*p* < 0.05) ADG, feed efficiency and day 39 body weight compared to the basal group. Supplementation with Se-enriched mushroom powder, at all inclusion levels, increased (*p* < 0.05) the abundance of *Prevotellaceae* and *Prevotella*, decreased (*p* < 0.05) the abundance of *Sporobacter* and increased (*p* < 0.05) the expression of *SELENOP* in the jejunum compared to the basal group. *Lactobacillaceae* and *Lactobacillus* was increased (*p* < 0.05) in 0.15 SeMP and 0.3 SeMP pigs compared to the basal group. Selenium deposition in muscle and liver tissue increased (*p* < 0.001) as a function of inclusion level while pigs supplemented with 0.3 ppm organic Se (0.3 SeMP) had an increase (*p* < 0.05) in total Se in the muscle compared to pigs supplemented with 0.3 ppm inorganic Se (basal). In conclusion, 0.3 SeMP supplementation led to positive effects on faecal scores and had similar pig performance compared to ZnO in Period 1, while the addition of 0.3 ppm selenite to 0.3 SeMP (0.6 SeMP/Sel) in Period 2 led to enhanced pig performance and aspects of gastrointestinal health.

## 1. Introduction

In commercial pig production systems, weaning can negatively impact immunity, gut health, growth and feed efficiency of pigs [1]. Post-weaning intestinal dysfunction is characterised by the upregulation of pro-inflammatory cytokines, atrophy of the small intestinal architecture, the proliferation of pathogenic bacteria [2] and the onset of post-weaning diarrhoea [3]. To help alleviate the challenges associated with weaning, pharmacological levels of zinc oxide (ZnO) have been supplemented to the diets of post-weaned pigs to enhance growth and reduce the proliferation of pathogenic bacteria [4]. However, due to the long-term accumulation of zinc in soils and its association with antimicrobial resistance [5], the EU has begun phasing out the use of pharmacological levels of ZnO, with its complete ban by 2022 (Commission Implementing Decision of 26 June 2017, C(2017) 4, 529 Final). Thus, there is an urgent requirement to develop alternative feed additives to prevent the post-weaning growth check and intestinal dysfunction in the absence of pharmacological levels of ZnO.

Selenium (Se) is an essential micronutrient which exerts various functions in enhancing antioxidant capacity, immunity, growth and meat quality in pigs [6]. Selenium is differentiated into either inorganic or organic sources which are commonly added to animal diets for enhancing growth performance and meat quality. However, inorganic selenium supplementation can have some disadvantages, such as toxicity, negative interaction with other minerals and have a low transfer efficiency to animal products [7] whilst organic sources of selenium are more bioavailable as it is readily absorbed in the digestive tract and has a higher threshold for toxicity compared to inorganic sources [8,9,10]. Dietary supplementation of organic Se, such as selenium-enriched yeast, could potentially help minimise post-weaning adversities as it has been shown to enhance antioxidant capacity, enhance immune function and suppress the inflammatory response in weaned pigs (0.25 ppm Se) [11] and also increases the immunoglobulin serum levels in finisher pigs (0.25 ppm Se) [12]. According to the National Research Council, 0.2–0.3 mg/kg (ppm) of added Se should meet the requirement for weaned pigs, with the maximum amount of added Se allowed in swine diets being 0.5 mg/kg of feed [13]. However, the supplementation of 0.7 mg/kg selenomethionine to post-weaned pig diets increased feed efficiency [14], suggesting that, in the absence of in-feed medication and ZnO, a higher Se inclusion level is required to enhance health, growth and antioxidant capacity of pigs.

Mushrooms are a rich natural source of bioactive metabolites, such as phenolic compounds and polysaccharides, including β-glucans [15]. Beta-glucans exhibit biological activity, such as anti-inflammatory, antioxidant and immunomodulatory effects [16,17,18]. Beta-glucans present in mushrooms can act as a prebiotic by positively influencing the gut microbiota through increased abundance of *Lactobacillus* and *Bifidobacteria* in broiler chickens [19] and turkey poults [20]. When irrigated with inorganic sodium selenite, *Agaricus bisporus* mushrooms can become rich in organic Se, namely selenocystine [21]. The antioxidant potential of mushrooms, due to their ability to scavenge free radicals [22], along with the antioxidant role of selenium [23,24], may perhaps increase the antioxidant status of the weaned pig, thus enhancing its immunomodulatory status. Therefore, *Agaricus bisporus* mushrooms offer a unique opportunity to naturally incorporate both β-glucans and organic selenium into the diet of pigs as well as being a natural, sustainable and cost-effective source of selenium.

Hence the objective of this study was to determine the optimum Se level, through the use of Se-enriched mushrooms and selenite, in weaned pig diets to enhance intestinal health, performance and antioxidant capacity. It was hypothesised that Se-enriched mushroom powder supplementation to the diet of post-weaned pigs would enhance gastrointestinal health, pig performance and antioxidant capacity, but may be influenced by Se inclusion level and time post-weaning.

## 2. Materials and Methods

All experimental procedures described in this work were approved under University College Dublin Animal Research Ethics Committee (AREC-20-22-O’Doherty) and conducted in accordance with Irish legislation (SI no. 543/2012) and the EU directive 2010/63/EU for animal experimentation. All efforts were taken to minimise pain and discomfort to the animal while conducting these experiments.

### 2.1. The Newly Weaned Pig (Period 1; Day 1–21)

#### 2.1.1. Experimental Design and Animal Management

The experiment had a complete randomised block design and consisted of the following five experimental groups: basal (basal + selenite (0.3 ppm Se)); ZnO (basal + selenite (0.3 ppm Se) + ZnO); 0.15 SeMP (basal + SeMP (0.15 ppm Se)); 0.3 SeMP (basal + SeMP (0.3 ppm Se)); 0.6 SeMP/Sel (basal + SeMP (0.3 ppm Se) + selenite (0.3 ppm Se)). The 0.15 SeMP group contained an additional 3.25 g/kg of plain mushroom powder and 3.25 g/kg of Se-enriched mushroom powder to maintain the same level of β-glucans across all mushroom diets. One hundred and twenty pigs (progeny of Meatline boars × (Large White × Landrace sows)) with an average weaning weight of 6.7 kg (SD 0.84 kg) were sourced from a commercial pig farm at weaning (28 days of age) and were penned in groups of three. The pigs were blocked by weaning weight, sex and litter of origin and within each block assigned to one of five dietary groups for the duration of the experiment (eight replicates/experimental group).

The ZnO (Cargill, Naas, Ireland) was included at 3100 mg ZnO/kg feed and contained 80% zinc, resulting in an inclusion level of 2500 mg Zn/kg of feed. The mushroom powders were sourced from Monaghan Mushroom (Tyholland, Co., Monaghan, Ireland) and were added at a rate of 6.5 g/kg to achieve a β-glucan content of 650 mg/kg across all mushroom groups (10% β-glucans). The *Agaricus bisporus* mushrooms were irrigated with sodium selenite as previously described by Maseko, et al. [21] to obtain an organic Se concentration of 45.8 mg/kg of mushroom powder in the form of selenocysteine. The diets were formulated to contain similar levels of net energy (10.6 MJ/kg) and standardised ileal digestible lysine (13.0 g/kg). The levels of amino acids were formulated to meet or exceed the requirements of the NRC (2012). All diets were milled on site and fed in meal form for the duration of the experiment. The ingredient composition and analysis of the diets are shown in Table 1.

#### 2.1.2. Housing and Animal Management

The pigs were housed in groups of three on fully slatted pens (1.68 × 1.22 m). They were weighed at the beginning of the experiment (d0; day of weaning) and on days 7, 14 and 21. The ambient environmental temperature within the house was thermostatically controlled at 30 °C for the first 7 days and then reduced by 2 °C each week, and the humidity was maintained at 65%. Feed in meal form and water were available ad libitum from four-space feeders and nipple drinkers. Faecal scores were recorded twice daily from individual pens from day 0 until day 21 by the same operator on a scale ranging from 1 to 5. The following scoring system was used: 1 = hard, firm faeces; 2 = slightly soft faeces; 3 = soft, partially formed faeces; 4 = loose, semi-liquid faeces; and 5 = watery, mucous-like faeces.

### 2.2. Physiological Effects of Selenium-Enriched Mushroom Powder (Period 2; Day 21–39)

Due to common practice of ZnO removal from the diet after the initial post-weaning period, the ZnO experimental group was removed from the experiment on day 21. The remaining pigs continued on their respective diet described in Period 1. Pigs were weighed at the beginning of the experiment (day 21) and at the end of the experiment (day 39). The housing and animal management were as described in Period 1, apart from the ambient environmental temperature, which was maintained at 24 °C for the duration of the experiment and the faecal scores, which were no longer recorded.

#### 2.2.1. Sample Collection

On day 39, eight pigs per experimental group (one pig/pen) received a lethal injection with pentobarbitone sodium (Euthatal Solution, 200 mg/mL; Merial Animal Health, Harlow, UK) at a rate of 0.71 mL/kg body weight to the cranial vena cava to humanely euthanise the animals. Euthanasia was performed by a competent person in a separate room away from sight and sound of the other pigs. The entire gastrointestinal tract was immediately removed. Sections from the duodenum (10 cm from the stomach), the jejunum (60 cm from the stomach) and the ileum (15 cm from the caecum) were excised and fixed in 10% neutral-buffered formalin (Teomics^®^, Houston, TX, USA). Digesta samples (approximately 10 g) from the caecum were aseptically collected into sterile containers (Sarstedt, Wexford, Ireland) and immediately frozen for subsequent 16 s rRNA sequencing and volatile fatty acid (VFA) analysis. In addition, tissue samples were taken from the duodenum, jejunum and ileum to measure the gene expression of cytokines, digestive enzymes, nutrient transporters, mucins, tight junctions and appetite regulators using qPCR. Tissue sections of 1 cm^2^ from the duodenum, jejunum and ileum were cut out, emptied by dissecting them along the mesentery and rinsed using sterile phosphate buffer saline (PBS) (Oxoid, Basingstoke, UK). The tissue sections were stripped of the overlying smooth tissue before storage in 5 mL RNAlater^®^ solution (Applied Biosystems, Foster City, CA, USA) overnight at 4 °C. The RNAlater^®^ was removed before storing the samples at −80 °C. Meat (muscle) and liver samples were taken for antioxidant analysis.

#### 2.2.2. Gut Morphological Analysis

Preserved duodenal, jejunal and ileal tissue samples were prepared using standard paraffin-embedding techniques. The samples were sectioned at a thickness of 5 μm and stained with haematoxylin–eosin. Villus height (VH) and crypt depth (CD) were measured in the stained sections (4 × objective) using a light microscope fitted with an image analyser (Image-Pro Plus; Media Cybernetics, Oxford, UK). Measurements of fifteen well-orientated and intact villi and crypts were taken for each segment. The VH was measured from the crypt–villus junction to the tip of the villus, and CD was measured from the crypt–villus junction to the base. Results are expressed as mean VH or CD in μm.

#### 2.2.3. Gene Expression

##### RNA Extraction and cDNA Synthesis

Total RNA was extracted from duodenal, jejunal, ileal and colonic tissue using TRI Reagent (Sigma-Aldrich, St. Louis, MO, USA) according to the manufacturer’s instructions. The crude RNA extract was further purified using the GenElute™ Mammalian Total RNA Miniprep kit (Sigma-Aldrich, St. Louis, MO, USA) according to the manufacturer’s instructions. A DNase removal step was included using an on-Column DNase 1 Digestion Set (Sigma-Aldrich). The total RNA was quantified using a Nanodrop-ND1000 Spectrophotometer (Thermo Scientific, Waltham, MA, USA) and the purity was assessed by determining the ratio of the absorbance at 260 nm and 280 nm. The RNA integrity was assessed using an Agilent 2100 Bioanalyzer using an RNA 6000 Nano LabChip kit (Agilent Technologies, Santa Clara, CA, USA). All samples had a 260:280 ratio > 2.0 and an RNA integrity number (RIN) > 8.0. The total RNA (2 μg) was reverse transcribed using a High-Capacity cDNA Reverse Transcription Kit (Applied Biosystems, Waltham, MA, USA) and oligo (dT) primers in a final reaction volume of 40 μL, according to the manufacturer’s instructions. The cDNA was then adjusted to a volume of 360 μL with nuclease-free water.

##### Quantitative PCR

The quantitative PCR (qPCR) reaction mix (20 μL) contained GoTaq qPCR Master Mix (10 μL) (Promega, Madison, WI, USA), forward and reverse primers (1.2 μL) (5 μM), nuclease-free water (3.8 μL) and cDNA (5 μL). All qPCR reactions were performed in duplicate on the 7500 ABI Prism Sequence detection System (Applied Biosystems, Foster City, CA, USA). The cycling conditions included a denaturation step of 95 °C for 10 min followed by 40 cycles of 95 °C for 15 s and 60 °C for 1 min. All primers were designed using the Primer Express Software (Applied Biosystems, Foster City, CA, USA) and synthesised by MWG Biotech UK Ltd. (Milton Keynes, UK) and are presented in Table 2. Dissociation curves were generated to confirm the specificity of the resulting PCR products. The qPCR assay efficiencies were established by plotting the threshold cycle (Ct) values derived from 4-fold serial dilutions of cDNA against their arbitrary quantities and only assays exhibiting 90–110% efficiency and single products were used in this study. Normalised relative quantities were obtained using the qbase PLUS software (Biogazelle, Ghent, Belgium) from stable reference genes; *ACTB*, *H3F3A* and *YWHAZ* for the duodenum; *H3F3A* and *YWHAZ* for the jejunum; and *ACTB* and *H3F3A* for the ileum. These genes were selected as reference genes based on their M value (<1.5) generated by the GeNorm algorithm within GeNorm. The genes analysed in the current study are as follows: *SLC15A1* (previously known as *PEPT1*); *FABP2*; *SLC5A1* (previously known as *SGLT1*); *SLC2A2* (previously known as *GLUT2*); *SLC2A5* (previously known as *GLUT5*); *CCK*; *PPY*; *GLP1*; *NPY*; *TNF*; *CXCL8* (previously known as *IL8*); *IL6*; *IL10*; *IL17*; *IFNG*; *MUC1*; *MUC2*; *TLR4*; *CLDN1*; *CLDN3*; *SELENOP*; *TXNRD1*; *DI01*; *ACTB*; *H3F3A*; *YWHAZ*. The qbase PLUS software uses a generalized model of the delta-delta-Ct approach, thereby supporting the use of gene-specific amplification efficiencies and normalisation with multiple reference genes. All formulas of this model are detailed in Hellemans et al. [26].

#### 2.2.4. Microbial Analysis

##### Microbial DNA Extraction

Microbial genomic DNA was extracted from the pigs caecal digesta samples using a QIAamp PowerFecal Pro DNA stool kit (Qiagen, Crawley Down, UK) according to the manufacturer’s instructions. The quantity and quality of DNA were assessed using a Nanodrop ND-1000 Spectrophotometer (Thermo Scientific, Wilmington, DE, USA).

##### Illumina Sequencing

High-throughput sequencing of the V3-V5 hypervariable region of the bacterial 16S rRNA gene was performed on an Illumina MiSeq platform according to their standard protocols (Eurofins, Genomics, Ebersberg, Germany). Briefly, the V3–V5 region was PCR-amplified using universal primers containing adapter overhang nucleotide sequences for forward and reverse index primers. Amplicons were purified using AMPure XP beads (Beckman Coulter, Indianaopolis, IN, USA) and set up for the index PCR with Nextera XT index primers (Illumina, San Diego, CA, USA). The indexed samples were purified using AMPure XP beads, quantified using a fragment analyser (Agilent, Santa Clara, CA, USA), and equal quantities from each sample were pooled. The resulting pooled library was quantified using the Bioanalyzer 7500 DNA kit (Agilent, Santa Clara, CA, USA) and sequenced using the v3 chemistry (2 × 300 bp paired-end reads).

##### Bioinformatics

The bioinformatic analyses of the resulting sequences were performed by Eurofins Genomics (Eberberg, Germany) using the open source software package (version 1.9.1) Quantitative Insights into Microbial Ecology (Qiime) [27]. All raw reads passing the standard Illumina chastity filter were demultiplexed according to their index sequences (read quality score >30). The primer sequences were clipped from the starts of the raw forward and reverse read. If primer sequences were not perfectly matched, read pairs were removed to retain only high-quality reads. Paired-end reads were then merged if possible, to obtain a single, longer read that covered the full target region using the software FLASH 2.2.00 [28]. Pairs were merged with a minimum overlap size of 10 bp to reduce false-positive merges. The forward read was only retained for the subsequent analysis steps when merging was not possible. Merged reads were quality filtered according to the expected length and known length variations in the V3–V5 region (ca. 445 bp). The ends of retained forward reads were clipped to a total read length of 285 bp to remove low quality bases. Merged and retained reads containing ambiguous bases were discarded. The filter reads (merged and quality clipped retained forward reads) were used for the microbiome profiling. Chimeric reads were identified and removed based on the de-novo algorithm of UCHIME [29], as implemented in the VSEARCH package [30]. The remaining set of high-quality reads was processed using minimum entropy decomposition (MED) to partition reads to operational taxonomic units (OTU) [31,32]. DC-MEGABLAST alignments of cluster representative sequences to the NCBI nucleotide sequence database were performed for taxonomic assignment (from phylum to species) of each OTU. A sequence identity of 70% across at least 80% of the representative sequence was the minimal requirement for considering reference sequences. Abundances of bacterial taxonomic units were normalised using lineage-specific copy numbers of the relevant marker genes to improve estimates [33].

The normalised OTU table combined with the phenotype metadata and phylogenetic tree comprised the data matrix. This matrix was then put into the phyloseq package within R (http://www.r-project.org; version 3.5.0, accessed on 25 March 2022). Differential abundance testing was performed on tables extracted from the phyloseq object at phylum, family and genus levels. The model assessed the effect of ‘group,’ with the individual pig being the experimental unit. Eight pigs per group were used for the statistical analysis of the relative bacterial abundances.

#### 2.2.5. Volatile Fatty Acids Analysis

The VFA concentrations in caecal digesta were determined using gas liquid chromatography (GLC) according to the method described by Clarke, et al. [34]. A 1 g sample of digesta was diluted with distilled water (2.5 × weight of sample) and centrifuged at 1400× *g* for 10 min using a Sorvall GLC-2B laboratory centrifuge (DuPont, Wilmington, DE, USA). The supernatant (1 mL) and internal standard (1 mL; 0.05% 3-methyl-n-valeric acid in 0.15 M oxalic acid dihydrate) were mixed with 3 mL of distilled water. The mixture was centrifuged at 500× *g* for 10 min and the supernatant was filtered through 0.45 TFE (polytetrafluoroethylene) syringe filter into a chromatographic sample vial. An injection volume of 1 μL was injected into a Varian 3800 GC (Ottawa, ON, Canada) equipped with an EC™ 1000 Grace column (15 m × 0.53 mm I.D) with 1.20 μm film thickness. The temperature programme set was 75–95 °C increasing by 3 °C/min, and 95–200 °C increasing by 20 °C/min, which was held for 0.50 min. The detector and injector temperature were 280 °C and 240 °C, respectively, while the total analysis time was 12.42 min.

#### 2.2.6. Feed Analysis

The feed samples were milled through a 1 mm screen (Christy and Norris Hammer Mill, Ipswich, UK). The dry matter content of the feed was determined after drying overnight at 104 °C. Ash content was determined after ignition of a weighted sample in a muffle furnace (Nabertherm) at 550 °C for 6 h. The gross energy content was determined using an adiabatic bomb calorimeter (Parr Instruments, St, Moline, IL, USA). The nitrogen content was determined using the LECO FP 528 instrument (Leco Instruments, Stockport, UK Ltd.). The neutral-detergent fibre content was determined according to Van Soest, et al. [35] using the Ankom 220 Fibre Analyser (Ankom^TM^ Technology, New York, NY, USA). The total glucans of the MP were determined using the kit K-YBGL, purchased from Megazyme (Bray, Co Wicklow, Ireland), following the manufacturer’s recommendations, and as previously described [36]. The selenium content was measured by Eurofins Food Testing UK Ltd. (Wolverhampton, United Kingdom) using the selenium in food method. All samples were measured in duplicate.

#### 2.2.7. Antioxidant Activity Analysis

Five grams of muscle/liver sample were homogenised (Stomacher 400 circulator, Steward Ltd., Fareham, UK) with 50 mL phosphate buffer (0.05 M, pH 7) for 3 min. The resulting homogenate was centrifuged at 4600 rpm at 4 °C for 15 min (Rotanta 460 R, Zentrifugen, Hettich, Kirchlengern, Germany) and the supernatant was collected for further analysis. The collected meat supernatant was tested for the determination of total antioxidant status (TAS) by 2,2-diphenyl-1-picrylhydrazyl radical scavenging capacity (DPPH) and ferric reducing antioxidant power (FRAP) assays.

##### DPPH Free Radical Scavenging Assay

The DPPH radical scavenging assay was carried out by adding 1 mL of DPPH to 1 mL of sample. This was incubated at room temperature for 20 min and then centrifuged at 2500 rpm for 15 min and read on the spectrometer at 515 nm. The amount of scavenging can be quantified by the change in colour from a deep violet to a yellow [37].

$$\mathrm{Scavenging}\;\mathrm{capacity}\;(\%)=\left(\frac{A\;\mathrm{control}\;-\;A\;\mathrm{sample}}{A\;\mathrm{control}\;}\right)\times 100$$

where ‘A control’ is the absorbance of the control (DPPH solution without sample) and A sample is the absorbance of the test sample (DPPH solution plus test sample).

##### FRAP Assay

The FRAP assay was carried out to assess the reducing power of the sample according to the method reported by Benzie and Strain [38], with some modifications by Rajauria, et al. [39]. Trolox (6-hydroxy-2, 5, 7, 8-tetrametmethylchroman-2-carboxyl acid) was used as a standard, and the results were expressed as mg of Trolox equivalents/kg muscle/liver sample. Increased absorbance of the reaction mixture indicated higher reducing power of the supplements in the meat samples.

#### 2.2.8. Total Selenium Analysis

Total selenium content was determined in muscle and liver samples according to the method reported by Bierla, et al. [40]. Briefly, samples were freeze dried (<500 µm) and digested on a hot plate at 65 °C with a mixture of nitric acid and hydrogen peroxide for 6 h. Total selenium was measured using size-exclusion chromatography-inductively couples plasma mass spectroscopy (ICP MS).

#### 2.2.9. Statistical Analysis

All data on growth performance, gastrointestinal morphology, gene expression, antioxidant analysis and VFA were checked for normality using the univariate procedure of Statistical Analysis Software (SAS) 9.4 and transformed, if required. The general linearized model (GLM) procedure within SAS was used to analyze the data on growth performance, gastrointestinal morphology, gene expression (Bonferroni adjusted *p* < 0.05), antioxidant analysis and VFA concentrations. The model examined the effects of treatment, using weight at weaning as a covariate. Faecal scores were averaged for every three days for the first 21 days and analysed using the PROC MIXED procedure of SAS. The model examined the effect of treatment, time and the associated interaction and using weight at weaning as a covariate The microbiome data were analysed using PROC GLIMMIX. Results are presented using Benjamini–Hochberg (BH) adjusted *p*-values. The pen was the experimental unit for growth performance and faecal scores, while the individual pig was the experimental unit for gastrointestinal morphology, gene expression, antioxidant analysis, microbiome and VFA data. The results are presented as least-square means with their standard errors. The probability level that denotes significance is *p* < 0.05.

## 3. Results

### 3.1. Period 1 (Day 1–21 Post-Weaning)

#### Performance and Faecal Scores

The effects of diet on average daily feed intake (ADFI), average daily gain (ADG), gain-to-feed ratio (G:F) and body weight up to day 21 post-weaning (Day 1–21) are presented in Table 3. Pigs supplemented with 0.15 SeMP had reduced (*p* < 0.05) ADG, ADFI and day 21 body weight compared to pigs supplemented with ZnO. Pigs supplemented with 0.3 SeMP had reduced (*p* < 0.05) ADFI compared to the basal and ZnO groups while pigs supplemented with 0.6 SeMP/Sel had a reduced (*p* < 0.05) ADFI compared to the basal group.

The effects of diet on faecal scores from days 1 to 21 post-weaning are presented in Table 3. Pigs supplemented with ZnO had reduced (*p* < 0.05) faecal scores compared to 0.15 SeMP, 0.6 SeMP/Sel and basal groups, while pigs supplemented with 0.3 SeMP had reduced (*p* < 0.05) faecal scores compared to the basal and 0.6 SeMP/Sel groups.

### 3.2. Period 2 (Day 21–39 Post-Weaning)

#### 3.2.1. Performance

The effects of diet on ADFI, ADG, G:F and body weight from days 21 to 39 are presented in Table 4. Pigs supplemented with 0.6 SeMP/Sel had increased (*p* < 0.05) ADG compared to all other dietary groups and had increased (*p* < 0.05) G:F and day 39 body weight compared to the basal group. There was no effect (*p* > 0.05) of diet on ADFI.

#### 3.2.2. Small Intestinal Morphology on Day 39

The effects of diet on the small intestinal morphology are presented in Table 5. There was no effect (*p* > 0.05) of diet on villus height, crypt depth or villus height to crypt depth ratio in the duodenum, jejunum or ileum.

#### 3.2.3. Differential Bacterial Abundance Analysis on Day 39

The effects of diet on bacterial phylum, family and genus in caecal digesta are presented in Table 6a–c, respectively.

At phylum level, the relative abundance of Bacteroidetes was increased (*p* < 0.05) in 0.15 SeMP, 0.3 SeMP and 0.6 SeMP/Sel groups compared to the basal group, with 0.15 SeMP having the highest abundance.

At family level, the relative abundance of *Prevotellaceae* was increased (*p* < 0.05) in the 0.15 SeMP, 0.3 SeMP and 0.6 SeMP/Sel groups compared to the basal group, with 0.15 SeMP having the highest abundance. The relative abundance of *Lactobacillaceae* was increased (*p* < 0.05), while *Ruminococcaceae* was decreased (*p* < 0.05) in the 0.15 SeMP and 0.3 SeMP groups compared to the basal and 0.6 SeMP/Sel groups.

At genus level, the relative abundance of *Prevotella* was increased (*p* < 0.001) in the 0.15 SeMP, 0.3 SeMP and 0.6 SeMP/Sel groups compared to the basal group with, 0.15 SeMP having the highest abundance. *Clostridium* was decreased (*p* < 0.05) in the 0.15 SeMP and 0.6 SeMP/Sel groups compared to the basal group, while *Ruminococcus* was decreased (*p* < 0.05) in the 0.3 SeMP and 0.6 SeMP/Sel groups compared to the basal group. *Prevotellamassilia* and *Faecalibacterium* were increased (*p* < 0.05) in 0.6 SeMP/Sel compared to all other dietary groups. *Lactobacillus* was increased (*p* < 0.05) in the 0.15 SeMP and 0.3 SeMP groups compared to the 0.6 SeMP/Sel and basal groups. *Agathobacter* was increased (*p* < 0.05) in the 0.3 SeMP group compared to the 0.6 SeMP/Sel and basal groups, and *Sporobacter* was decreased (*p* < 0.05) in all dietary groups compared to the basal group.

#### 3.2.4. Volatile Fatty Acids on Day 39

The effects of diet on the concentrations of caecal VFAs are presented in Table 7. Surprisingly, there was no effect (*p* > 0.05) of diet on VFA concentrations.

#### 3.2.5. Gene Expression in the Small Intestine on Day 39

The effects of diet on the expression of genes related to appetite regulators, tight junctions, nutrient transporters, selenium transporters, cytokines and mucins in the duodenum, jejunum and ileum are presented in Table 8a, Table 8b and Table 8c, respectively.

There was no effect (*p* > 0.05) of diet on gene expression in the duodenum.

In the jejunum, *SELENOP* was upregulated (*p* < 0.05) in all dietary groups compared to the basal group, with 0.6 SeMP/Sel having the highest abundance. *SGLT1* was upregulated (*p* < 0.05) in the 0.15 SeMP and 0.6 SeMP/Sel groups compared to the 0.3 SeMP and basal groups. *IFNG* was upregulated (*p* < 0.05) in the 0.15 SeMP group compared to the basal group.

In the ileum, *MUC1* was downregulated (*p* < 0.05) in the 0.15 SeMP and 0.3 SeMP groups compared to the basal and 0.6 SeMP/Sel groups.

#### 3.2.6. Total Antioxidant of Muscle and Liver on Day 39

The effects of diet on total antioxidant in the muscle and liver is presented in Table 9. There was no effect (*p* > 0.05) of diet on FRAP or DPPH assays in the muscle and liver.

#### 3.2.7. Total Selenium Content of Muscle and Liver on Day 39

The effects of diet on total selenium content in the muscle and liver is presented in Table 10. In the muscle, total selenium was increased (*p* < 0.001) in the 0.3 SeMP and 0.6 SeMP/Sel groups compared to the basal and 0.15 SeMP groups, with 0.6 SeMP/Sel having the highest concentration (*p* < 0.05) compared to all other groups. In the liver, total selenium was increased (*p* < 0.001) in the basal, 0.3 SeMP and 0.6 SeMP/Sel groups compared to the 0.15 SeMP group, with 0.6 SeMP/Sel having the highest concentration (*p* < 0.05) compared to all other groups.

## 4. Discussion

It was hypothesised that Se-enriched mushroom powder supplementation to the diet of post-weaned pigs would enhance gastrointestinal health, pig performance and antioxidant capacity, but may be influenced by Se inclusion level and time post-weaning. In Period 1, dietary supplementation with 0.15 SeMP reduced ADG, ADFI and day 21 body weight and increased faecal scores compared to ZnO supplementation. Supplementation with 0.3 SeMP during Period 1 resulted in positive effects on faecal scores and similar pig performance compared to ZnO supplementation, while also leading to the increased abundance of *Prevotella* and *Lactobacillus*, decreased abundance of *Sporobacter* and increased expression of *SELENOP* at the end of Period 2. Dietary supplementation with 0.6 SeMP/Sel during Period 2 led to increased ADG, G:F and day 39 body weight, while also increasing the abundance of *Prevotella* and *Faecalbacterium*, decreasing the abundance of *Sporobacter* and increasing the expression of *SELENOP*.

Faecal scoring has been considered as an indicator of gut health [41]. According to the scoring system used in the current study, faecal scores between 3 and 5 are representative of diarrhoea, and all groups in this study had faecal scores between 2 and 3, indicating that pigs were not under major immunological or microbiological stress. These healthy scores are likely due to the good hygiene and husbandry practices observed in research facilities [42,43]. However, it is worth noting that pigs supplemented with 0.3 SeMP had similar faecal scores and performance results compared to pigs supplemented with ZnO, perhaps suggesting that 0.3 ppm Se supplementation is the desired inclusion level of organic Se for the immediate post-weaned period. Interestingly, the Se inclusion level of 0.6 ppm (0.6 SeMP/Sel) resulted in increased faecal scores compared to 0.3 SeMP and ZnO groups during the first 21 days post-weaning. However, the faecal scores were still within the normal healthy range (<3). During days 21 to 39, pigs supplemented with 0.6 SeMP/Sel had increased daily gain, gain-to-feed ratio and day 39 body weight. Similarly, Cao, Guo, Zhang, Dong and Gong [14] concluded that dietary supplementation with 0.7 ppm selenomethionine for 42 days post-weaning increased feed efficiency in post-weaned pigs, perhaps suggesting that a higher Se inclusion in the diet of pigs, after the initial post-weaning period, has the potential to enhance pig growth performance.

Maximising feed intake during the post-weaning period is critical, as low feed intake in newly weaned pigs negatively affects nutrient uptake and utilisation, leading to undesired physiological, morphological and microbial changes in the gastrointestinal tract (GIT) [3]. In Period 1, Se-enriched mushroom powder supplementation, at all inclusion levels, reduced feed intake compared to basal fed pigs. While the aim of the study was not to explore the mechanism behind the reduced feed intake, it may be due to the mushroom powder having a satiety effect and/or reduced palatability. Food components present in mushroom powder, such as the non-digestible polysaccharide chitin, may increase the satiety effect, leading to a reduction in feed intake. Chitin supplementation, through its deacetylated form, reduces feed intake in both mice [44] and pigs [45,46]. Mushrooms contain a spectrum of anti-nutritional compounds, such as tannins, alkaloids, saponins and phytases [47], which, due to their astringent taste, may negatively affect their palatability, resulting in a reduced feed intake [48]. Nevertheless, while 0.3 SeMP and 0.6 SeMP/Sel reduced feed intake compared to the basal diet during Period 1, there was no adverse effects on average daily gain or feed efficiency.

Dietary intervention with natural bioactives can positively influence the microbial composition of the large intestine of pigs, leading to enhanced animal health and performance and inhibiting pathogenic colonisation. Dietary supplementation with Se-enriched mushroom powder, at all inclusion levels, increased the relative abundance of the family *Prevotellaceae* and genus *Prevotella*. Research has shown *Prevotella* to be associated with fibre intake and the degradation of carbohydrates [49], with a higher abundance of *Prevotella* being associated with improved weight gain and feed efficiency and decreased incidence of diarrhoea [50,51,52]. These findings suggest that *Prevotella* promotes pig growth performance and health and that the high β-glucan content (650 mg/kg) present in the mushroom-based diets led to the increased abundance.

Within the phylum Firmicutes, Se-enriched mushroom powder supplementation decreased the abundance of *Sporobacter,* while pigs supplemented with 0.15 SeMP and 0.3 SeMP had a greater abundance of the family *Lactobacillaceae* and genus *Lactobacillus*. Recent research has suggested that *Sporobacter* may have a detrimental impact on pig gut health [53], thus a lesser abundance is optimal for enhanced pig health. *Lactobacillus* species can enhance GIT health through the competitive exclusion of pathogens, antioxidant activities and aiding in immune system regulation [54,55]. We suspect the increase in *Lactobacillaceae* and *Lactobacillus* is due to the presence of organic Se (selenocystine) in the mushroom powder, as a negative effect was associated with the addition of selenite in both the 0.6 SeMP/Sel and basal groups (both groups containing 0.3 ppm selenite). Dietary supplementation with 0.6 SeMP/Sel increased the relative abundance of *Faecalibacterium,* which has been associated with increased intestinal health in weaned pigs [56], while *Faecalibacterium prausnitzii* is considered a promising probiotic in human medicine due to its use in treating inflammatory-related diseases [57]. These results indicate that Se-enriched mushroom powder supplementation positively influenced the microbial composition of post-weaned pigs. The exact mode of action of Se-enriched mushroom powder on the caecal microbiome was beyond the scope of this study, but it warrants further exploration. Despite selenium-enriched mushroom powder supplementation increasing the abundance of *Prevotella*, *Lactobacillus* and *Faecalbacterium*, there were no effects on VFA production or proportions.

Gastrointestinal homeostasis is critical to the health of weaned pigs and the disruption to this gives rise to intestinal inflammation. There was minimal change in the expression of cytokines between groups in this experiment and as a result, there were no effects on the epithelial barrier function or intestinal architecture.

The current results show that the highest Se deposition in both the muscle and liver was found in pigs fed 0.6 ppm Se, indicating that Se was increased as a function of inclusion level. Interestingly, pigs supplemented with 0.3 ppm organic Se (0.3 SeMP) had an increase in total Se in the muscle compared to pigs supplemented with 0.3 ppm inorganic Se (basal). Although there are several selenoproteins in vertebrae, the selenoproteins measured in this study are the best known [58]. The expression of *SELENOP*, a selenium transporter, was upregulated in the jejunum of pigs supplemented with Se-enriched mushroom powder, irrespective of Se inclusion level, suggesting that the bioavailability, absorption and utilisation of organic Se is higher compared to inorganic Se sources. In the muscle, there was no difference in Se deposition between pigs supplemented with 0.3 ppm inorganic Se (basal) and pigs supplemented with 0.15 organic Se (0.15 SeMP), suggesting that organic Se has a greater ability to deposit in muscle compared to inorganic Se. The differences in total Se deposition between the muscle and liver may perhaps be due to the different roles they play in Se metabolism. The liver is a main organ for selenoprotein synthesis, Se metabolism and Se transport to other tissues, while the muscle is a major organ for Se storage [59,60]. Selenium plays an important role in antioxidant activity, as it can prevent peroxidation and protect the immune cells and tissues from damage caused by the free radicals [23,24]. It was anticipated that antioxidant activity would increase as a function of Se inclusion level. However, there was no effect on FRAP or DPPH assays in both the muscle and liver at all Se inclusion levels.

## 5. Conclusions

Selenium-enriched mushroom powder supplementation at Se inclusion level of 0.3 ppm led to positive effects on faecal scores and similar post-weaning pig performance compared to ZnO supplementation during the first 21 days post-weaning. Selenium-enriched mushroom powder supplementation in combination with selenite, at a Se inclusion of 0.6 ppm, increased ADG and feed efficiency during days 21 to 39 and increased day 39 body weight. Selenium-enriched mushroom powder supplementation increased the abundance of *Prevotella*, *Lactobacillus* and *Faecalbacterium*, decreased the abundance of *Sporobacter* and increased the expression of *SELENOP,* while Se deposition in muscle and liver tissue increased as a function of inclusion level. These results suggest that 0.3 ppm organic Se supplementation is satisfactory for the immediate post-weaning period in terms of maintaining performance and enhancing faecal scores. However, a higher level of Se (0.6 ppm), led to enhanced pig performance while positively modulating intestinal microbiota after the initial post-weaning period. Selenium-enriched mushroom powder supplementation to pigs is therefore a novel and sustainable way to incorporate selenium and β-glucans into the diet of weaned pigs.

## Figures and Tables

**Table 1:** Ingredient and chemical composition of all diets.

	Treatments				
Ingredients (g/kg Unless Otherwise Stated)	Basal	ZnO	0.15 SeMP	0.3 SeMP	0.6 SeMP/Sel
Sodium selenite (mg/kg)	6.5	6.5	0	0	6.5
Mushroom Powder	0	0	3.25	0	0
Se Mushroom Powder	0	0	3.25	6.5	6.5
Wheat	355.4	352.1	348.9	348.9	348.9
Full-fat soya bean	170	170	170	170	170
Soya bean meal	105	105	105	105	105
Whey powder (90%)	50	50	50	50	50
Zinc oxide	0	3.3	0	0	0
Soya oil	30	30	30	30	30
Soya concentrate	65	65	65	65	65
Flaked wheat	130	130	130	130	130
Flaked maize	70	70	70	70	70
Lysine-HCl	4	4	4	4	4
DI-Methionine	2	2	2	2	2
L-Threonine	1.8	1.8	1.8	1.8	1.8
Tryptophan	0.3	0.3	0.3	0.3	0.3
Sodium bicarbonate	2	2	2	2	2
Monocalcium phosphate	4	4	4	4	4
Vitamins and minerals ^a^	2.5	2.5	2.5	2.5	2.5
Calcium carbonate (limestone)	6	6	6	6	6
Salt	2	2	2	2	2
Analysed chemical analysis					
DM	899.0	899.5	897.5	898.1	898.1
NDF	99.0	98.7	99.5	99.3	99.3
GE (MJ/kg)	16.9	16.9	16.8	16.9	16.9
Ash	46.2	46.1	46.0	46.2	46.0
Crude fat	79.9	80.3	80.1	80.0	80.2
β-glucan * (mg/kg)	3.0	4.0	649.0	652.0	655.0
Crude fibre	28.0	28.0	28.2	28.3	28.1
Crude protein	208.0	208.3	208.5	208.5	208.4
Lysine (%) ^¥^	1.4	1.4	1.4	1.4	1.4
Methionine (%) ^¥^	0.5	0.5	0.5	0.5	0.5
Threonine (%) ^¥^	0.9	0.9	0.9	0.9	0.9
Methionine and cysteine (%) ^¥^	0.8	0.8	0.8	0.8	0.8
Tryptophan (%) ^¥^	0.3	0.3	0.3	0.3	0.3
Valine (g/kg) ^¥^	19.0	19.0	19.0	19.0	19.0
Lactose (g/kg) ^¥^	34.0	34.0	34.0	34.0	34.0
Selenium (mg/kg)	0.28	0.30	0.16	0.31	0.62

Abbreviations: ZnO, zinc oxide; 0.15 SeMP, 0.15 ppm Se (selenium-enriched mushrooms); 0.3 SeMP, 0.3 ppm Se (selenium-enriched mushrooms); 0.6 SeMP/Sel, 0.6 ppm Se (0.3 ppm selenium-enriched mushrooms + 0.3 ppm selenite); DM, dry matter; GE, gross energy; NDF, neutral detergent fibre. *Analysed for β(1-3)-(1-6) β-glucan ^¥^ Calculated for the tabulated nutritional composition [25]. ^a^ Provided (per kg diet): 25 mg Cu; 140 mg Fe; 47 mg Mn; 120 mg Zn; 0.6 mg I; 0.3 mg S; 1.8 mg retinol; 0.025 mg cholecalciferol; 67 mg tocopherol; 4 mg menaquinone; 0.01 mg cyanocobalamin; 2 mg riboflavin; 12 mg nicotinic acid; 10 mg pantothenic acid; 250 mg choline chloride; 2 mg thiamine; and 0.015 mg pyridoxine.

**Table 2:** Panel of porcine oligonucleotide primers used for real-time PCR.

Group	Gene	Accession No.	Forward Primer (5’-3’) Reverse Primer (5’-3’)	Amplicon Length (bp)
Immune response	*IL6*	NM 214399.1	F: GACAAAGCCACCACCCCTAA R: CTCGTTCTGTGACTGCAGCTTATC	69
	*CXCL8*	NM 213867.1	F: TGCACTTACTCTTGCCAGAACTG R: CAAACTGGCTGTTGCCTTCTT	82
	*IL10*	NM 214041.1	F: GCCTTCGGCCCAGTGAA R: AGAGACCCGGTCAGCAACAA	71
	*IL17*	NM 001005729.1	F: CCCTGTCACTGCTGCTTCTG R: TCATGATTCCCGCCTTCAC	57
	*INFG*	NM 213948.1	F: TCTAACCTAAGAAAGCGGAAGAGAA R: TTGCAGGCAGGATGACAATTA	81
	*TNF*	NM 214022.1	F: TGGCCCCTTGAGCATCA R: CGGGCTTATCTGAGGTTTGAGA	68
	*TLR4*	NM 001293317.1	F: TGCATGGAGCTGAATTTCTACAA R: GATAAATCCAGCACCTGCAGTTC	140
Tight junctions and Mucins	*MUC1*	XM 001926883.1	F: ACACCCATGGGCGCTATGT R: GCCTGCAGAAACCTGCTCAT	68
	*MUC2*	AK 231524	F: CAACGGCCTCTCCTTCTCTGT R: GCCACACTGGCCCTTTGT	70
	*CLND1*	NM 001244539.1	F: CTGGGAGGTGCCCTACTTTG R: TGGATAGGGCCTTGGTGTTG	72
	*CLND3*	NM 001160075.1	F: GAGGGCCTGTGGATGAACTG R: GAGTCGTACACTTTGCACTGCAT	65
Appetite regulators	*CCK*	NM 214237.2	F: GGACCCCAGCCACAGAATAA R: GCGCCGGCCAAAATC	61
	*PPY*	XM 005668763.1	F: CTCCTGATTCGGTTTGCAGAA R: GGACAGGAGCAGCAGGAAGA	61
	*GLP1*	NM 214237.2	F: CAGTGCAGAAATGGCGAGAA R: GGTGGAGCCTCAGTCAGGAA	61
	*NPY*	NM 001256367.1	F: CAGGCAGAGATACGGAAAACG R: TCCGTGCCTCTCTCATCAAG	71
Nutrient transporters	*FABP2*	NM 001031780.1	F:CAGCCTCGCAGACGGAACTGAA R:GTGTTCTGGGCTGTGCTCCAAGA	102
	*SLC2A2/GLUT2*	XM 001097417.1	F:CCAGGCCCCATCCCCTGGTT R:GCGGGTCCAGTTGCTGAATGC	96
	*SLC2A5/GLUT5*	XM 021095252.1	F:CCCAGGAGCCGGTCAAG R:TCAGCGTCGCCAAAGCA	60
	*SLC5A1/SGLT1*	NM 001164021	F: GGCTGGACGAAGTATGGTGT R: ACAACCACCCAAATCAGAGC	153
	*SLC15A1/PEPT1*	NM 214347.1	F:GGATAGCCTGTACCCCAAGCT R:CATCCTCCACGTGCTTCTTGA	73
Selenium transporters	*SELENOP*	NM 001134823.1	F:CAGGCCAGCTGATACCTGTGT R:TTAGAATATCCTTCTTTCTCCAGTTTTACTC	21
	*TXNRD1*	NM 214154.3	F:CACCGTGACGGACTCAAAACT R:GCTTGAGGCTGGTGACTTCAT	20
	*DIO1*	NM 001001627.1	F:GGCTCTGGGTGCTCTTTCAG R:CAGGAAACAATGTCATGAGCACTT	21
Reference genes	*ACTB*	AY550069.1	F: CAAATGCTTCTAGGCGGACTGT R: TCTCATTTTCTGCGCAAGTT	75
	*H3F3A*	NM 001014389.2	F: CATGGCTCGTACAAAGCAGA R: ACCAGGCCTGTAACGATGAG	136
	*YWHAZ*	XM 001927228.1	F: GGACATCGGATACCCAAGGA R: AAGTTGGAAGGCCGGTTAATTT	71

**Table 3:** Effect of dietary treatment on pig growth performance and faecal scores (Period 1; Day 1–21).

	Treatments *					SEM	*p*-Value
	Basal	ZnO	0.15 SeMP	0.3 SeMP	0.6 SeMP/Sel		
ADG (kg)	0.27 ^b^	0.27 ^b^	0.23 ^a^	0.26 ^a,b^	0.26 ^a,b^	0.013	0.017
ADFI (kg)	0.39 ^c^	0.38 ^b,c^	0.32 ^a^	0.35 ^a,b^	0.36 ^b^	0.008	<0.001
G:F	0.65	0.66	0.63	0.69	0.66	0.045	0.939
Weight (kg)	12.6 ^b^	12.5 ^b^	11.6 ^a^	12.2 ^a,b^	12.3 ^a,b^	0.266	0.034
Faecal score ^$^	2.57 ^c^	2.37 ^a^	2.52 ^b,c^	2.44 ^a,b^	2.61 ^c^	0.038	<0.001

Abbreviations: ZnO, zinc oxide; 0.15 SeMP, 0.15 ppm Se (selenium-enriched mushrooms); 0.3 SeMP, 0.3 ppm Se (selenium-enriched mushrooms); 0.6 SeMP/Sel, 0.6 ppm Se (0.3 ppm selenium-enriched mushrooms + 0.3 ppm selenite); ADG, average daily gain; ADFI, average daily feed intake; G:F, gain-to-feed ratio. ^a,b,c^ Mean values within a row with unlike superscript letters were significantly different (*p* < 0.05). * A total of eight replicates were used per experimental group (replicate = pen, three pigs/pen). ^$^ There was no treatment x time interaction (*p* > 0.05). (Least-square mean values with their standard errors).

**Table 4:** Effect of dietary treatment on pig growth performance (Period 2; Day 21–39).

	Treatments *				SEM	*p*-Value
	Basal	0.15 SeMP	0.3 SeMP	0.6 SeMP/Sel		
ADG (kg)	0.59 ^a^	0.62 ^a^	0.62 ^a^	0.67 ^b^	0.019	0.025
ADFI (kg)	0.90	0.90	0.91	0.93	0.023	0.772
G:F	0.67 ^a^	0.68 ^a,b^	0.68 ^a,b^	0.74 ^b^	0.025	0.049
Weight (kg)	22.9 ^a^	23.5 ^a,b^	23.5 ^a,b^	24.3 ^b^	0.342	0.046

Abbreviations: 0.15 SeMP, 0.15 ppm Se (selenium-enriched mushrooms); 0.3 SeMP, 0.3 ppm Se (selenium-enriched mushrooms); 0.6 SeMP/Sel, 0.6 ppm Se (0.3 ppm selenium-enriched mushrooms + 0.3 ppm selenite); ADG, average daily gain; ADFI, average daily feed intake; G:F, gain-to-feed ratio. ^a,b^ Mean values within a row with unlike superscript letters were significantly different (*p* < 0.05). * A total of eight replicates were used per experimental group (replicate = pen, three pigs/pen). (Least-square mean values with their standard errors).

**Table 5:** Effect of dietary treatment on villus height and crypt depth in the small intestine on day 39 post-weaning.

	Treatments *				SEM	*p*-Value
	Basal	0.15 SeMP	0.3 SeMP	0.6 SeMP/Sel		
Duodenum						
VH (µm)	306.55	329.24	312.07	337.51	14.587	0.414
CD (µm)	136.16	133.69	140.18	126.82	6.822	0.574
VH:CD	2.31	2.53	2.25	2.67	0.161	0.250
Jejunum						
VH (µm)	290.12	303.03	281.47	324.03	20.599	0.501
CD (µm)	138.30	146.86	137.90	132.50	8.778	0.715
VH:CD	2.13	2.07	2.04	2.47	0.117	0.056
Ileum						
VH (µm)	267.90	265.69	265.27	283.68	13.630	0.744
CD (µm)	134.41	127.89	127.05	130.98	6.127	0.827
VH:CD	2.03	2.08	2.12	2.17	0.112	0.843

Abbreviations: 0.15 SeMP, 0.15 ppm Se (selenium-enriched mushrooms); 0.3 SeMP, 0.3 ppm Se (selenium-enriched mushrooms); 0.6 SeMP/Sel, 0.6 ppm Se (0.3 ppm selenium-enriched mushrooms + 0.3 ppm selenite); VH, villus height; CD, crypt depth; VH:CD, villus height to crypt depth ratio. * A total of eight replicates were used per experimental group (replicate = pig). (Least-square mean values with their standard errors).

**Table 6:** Effect of dietary treatment on differential relative abundance of bacterial taxa at the (**a**) phylum level, (**b**) family level and (**c**) genus level in pig caecal digesta on day 39 post-weaning.

(a)						
	Treatments *				SEM	*p*-Value
	Basal	0.15 SeMP	0.3 SeMP	0.6 SeMP/Sel		
Bacteroidetes	9.808 ^a^	25.447 ^c^	18.144 ^b^	20.927 ^a,b^	1.546	<0.001
Actinobacteria	0.724	0.319	1.431	0.966	0.323	0.194
Firmicutes	83.194	72.091	77.637	76.413	3.167	0.116
Proteobacteria	1.052	0.381	1.342	0.800	0.331	0.315
(**b**)						
	Treatments *				**SEM**	***p*-Value**
	**Basal**	**0.15 SeMP**	**0.3 SeMP**	**0.6 SeMP/Sel**		
*Prevotellaceae*	9.860 ^a^	25.904 ^c^	18.707 ^b^	21.558 ^a,b^	1.564	<0.001
*Clostridiaceae*	9.914	6.367	8.464	6.568	1.000	0.059
*Selenomonadaceae*	0.175	0.477	0.443	0.362	0.216	0.752
*Oscillospiraceae*	0.796	0.324	0.269	0.307	0.226	0.364
*Hungateiclostridiaceae*	0.957	0.968	0.645	0.828	0.331	0.888
*Atopobiaceae*	0.317	0.236	0.964	0.610	0.253	0.252
*Lactobacillaceae*	1.218 ^a^	4.270 ^b^	3.285 ^b^	0.983 ^a^	0.541	0.001
*Ruminococcaceae*	43.546 ^b^	31.490 ^a^	34.813 ^a^	41.439 ^b^	2.210	0.002
*Lachnospiraceae*	18.879	21.841	22.902	17.265	1.619	0.080
*Eubacteriaceae*	2.489	2.380	1.417	3.266	0.553	0.177
*Acidaminococcaceae*	0.948	0.809	0.776	0.581	0.316	0.880
*Veillonellaceae*	0.574	0.780	1.083	1.580	0.357	0.263
*Erysipelotrichaceae*	1.127	0.840	0.619	0.816	0.330	0.736
*Streptococcaceae*	1.017	0.288	1.535	1.010	0.339	0.192
*Coriobacteriaceae*	0.269	0.111	0.560	0.413	0.201	0.549
*Campylobacteraceae*	0.118	0.190	0.612	0.278	0.189	0.357
*Rikenellaceae*	0.260	0.520	0.161	0.258	0.194	0.652
(**c**)						
	Treatments *				**SEM**	***p*-Value**
	**Basal**	**0.15 SeMP**	**0.3 SeMP**	**0.6 SeMP/Sel**		
*Blautia*	2.292	2.460	2.330	2.158	0.548	0.986
*Prevotella*	4.776 ^a^	18.365 ^c^	13.494 ^b^	13.851 ^b^	1.263	<0.001
*Clostridium*	9.446 ^b^	5.729 ^a^	7.852 ^a,b^	5.951 ^a^	0.960	0.037
*Dorea*	1.365	1.204	1.821	1.649	0.442	0.766
*Prevotellamassilia*	3.138 ^a^	3.813 ^a^	4.054 ^a^	6.550 ^b^	0.752	0.023
*Anaerovibrio*	0.124	0.347	0.447	0.365	0.201	0.703
*Coprococcus*	2.547	1.781	1.383	2.053	0.498	0.405
*Oscillibacter*	0.679	0.229	0.211	0.308	0.207	0.408
*Anaerobacterium*	0.818	0.795	0.611	0.584	0.301	0.922
*Olsenella*	0.315	0.238	0.987	0.615	0.255	0.234
*Lactobacillus*	1.235 ^a^	4.285 ^b^	3.334 ^b^	0.990 ^a^	0.544	0.001
*Faecalibacterium*	16.095 ^a^	14.448 ^a^	17.747 ^a^	24.937 ^b^	1.538	0.001
*Agathobacter*	5.053 ^a^	6.407 ^a,b^	9.051 ^b^	5.988 ^a^	0.924	0.027
*Fournierella*	1.226	1.404	2.140	1.491	0.449	0.496
*Anaerobium*	0.953	0.246	0.566	0.330	0.249	0.284
*Eubacterium*	2.507	2.384	1.437	3.298	0.555	0.176
*Butyrivibrio*	0.097	0.026	0.014	0.095	0.081	0.893
*Lachnobacterium*	0.430	0.754	0.407	0.389	0.252	0.735
*Phascolarctobacterium*	0.938	0.684	0.776	0.587	0.309	0.872
*Dialister*	0.380	0.517	0.628	1.231	0.294	0.250
*Butyricicoccus*	0.418	0.456	0.651	0.603	0.262	0.902
*Gemmiger*	9.255	6.655	8.219	9.187	1.037	0.301
*Megasphaera*	0.193	0.271	0.473	0.351	0.203	0.786
*Streptococcus*	1.016	0.289	1.561	1.020	0.341	0.181
*Roseburia*	2.964	4.660	4.400	2.164	0.672	0.058
*Mediterraneibacter*	0.805	1.049	0.628	0.674	0.319	0.808
*Pseudobutyrivibrio*	0.210	0.464	0.579	0.359	0.226	0.690
*Sporobacter*	9.496 ^b^	3.727 ^a^	3.872 ^a^	2.365 ^a^	0.758	<0.001
*Enorma*	0.270	0.112	0.427	0.203	0.175	0.705
*Agathobaculum*	0.204	0.163	0.357	0.566	0.200	0.557
*Ruminococcus*	5.343 ^b^	3.703 ^a,b^	2.500 ^a^	2.325 ^a^	0.658	0.013
*Oribacterium*	0.045	0.345	0.361	0.249	0.173	0.650
*Intestinimonas*	0.401	0.277	0.139	0.125	0.169	0.669
*Campylobacter*	0.121	0.191	0.618	0.280	0.190	0.353
*Alistipes*	0.163	0.380	0.166	0.259	0.176	0.810
*Pseudoflavonifractor*	1.361	0.509	0.114	0.450	0.258	0.055
*Kineothrix*	1.124	1.318	0.410	0.047	0.274	0.115

Abbreviations: 0.15 SeMP, 0.15 ppm Se (selenium-enriched mushrooms); 0.3 SeMP, 0.3 ppm Se (selenium-enriched mushrooms); 0.6 SeMP/Sel, 0.6 ppm Se (0.3 ppm selenium-enriched mushrooms + 0.3 ppm selenite). ^a,b,c^ Mean values within a row with unlike superscript letters were significantly different (*p* < 0.05). * A total of eight replicates were used per experimental group (replicate = pig). (Least-square mean values with their standard errors).

**Table 7:** Effect of dietary treatment on total volatile fatty acids (VFA) in caecal digesta on day 39 post-weaning.

	Treatments *				SEM	*p*-Value
	Basal	0.15 SeMP	0.3 SeMP	0.6 SeMP/Sel		
VFA (mmol/l digesta)						
Total	170.64	160.76	175.87	169.53	15.112	0.912
Acetate	126.24	109.84	123.51	122.70	12.120	0.788
Propionate	32.23	31.54	34.00	31.58	2.477	0.880
Butyrate	12.73	15.52	16.03	12.11	1.732	0.296
Isobutyrate	0.69	0.43	0.50	0.73	0.120	0.260
Isovalerate	1.26	2.06	0.64	1.07	0.436	0.165
Valerate	1.75	1.37	1.19	1.34	0.233	0.365
Branch	3.70	3.87	2.33	3.14	0.657	0.347

Abbreviations: 0.15 SeMP, 0.15 ppm Se (selenium-enriched mushrooms); 0.3 SeMP, 0.3 ppm Se (selenium-enriched mushrooms); 0.6 SeMP/Sel, 0.6 ppm Se (0.3 ppm selenium-enriched mushrooms + 0.3 ppm selenite). * A total of eight replicates were used per experimental group (replicate = pig). (Least-square mean values with their standard errors).

**Table 8:** Effect of dietary treatment on the expression appetite regulators, tight junctions, nutrient transporters, selenium transporters, cytokines and mucins in the (**a**) duodenum, (**b**) jejunum and (**c**) iluem on day 39 post-weaning.

(a)						
	Treatments *				SEM	*p*-Value
	Basal	0.15 SeMP	0.3 SeMP	0.6 SeMP/Sel		
Appetite regulators						
*PYY*	1.068	1.188	1.039	1.223	0.210	0.906
*GLP1*	0.676	1.017	0.653	0.933	0.300	0.758
*NPY*	0.864	0.953	0.944	1.448	0.176	0.096
*CCK*	1.128	1.307	1.049	1.406	0.196	0.557
Tight junctions						
*CLDN1*	1.374	1.289	1.027	0.921	0.249	0.535
*CLDN3*	0.813	1.596	1.495	1.335	0.268	0.214
Nutrient transporters						
*SLC2A2/GLUT2*	0.934	1.138	0.973	1.026	0.132	0.721
*SLC2A5/GLUT5*	0.998	1.182	1.312	1.252	0.264	0.859
*SLC15A1/PEPT1*	0.822	1.191	1.186	0.888	0.143	0.165
*FABP2*	0.784	1.280	1.196	0.977	0.140	0.085
Cytokines						
*IFNG*	0.930	1.132	1.126	0.963	0.210	0.858
*TNF*	1.037	1.073	1.071	0.923	0.089	0.590
*TLR4*	1.148	1.092	0.868	1.067	0.179	0.709
*IL10*	1.039	1.234	1.031	0.699	0.219	0.380
*IL6*	1.247	1.020	0.880	0.832	0.154	0.267
*IL17*	1.089	1.516	0.936	1.248	0.284	0.516
*CXCL8/IL8*	1.218	0.968	1.121	1.129	0.196	0.842
Mucins						
*MUC1*	1.104	1.063	1.462	1.421	0.292	0.671
*MUC2*	1.022	1.488	1.400	1.321	0.288	0.706
Selenium transporters						
*SELENOP*	1.218	1.089	0.872	0.959	0.219	0.709
*TXNRD1*	0.921	1.019	1.248	1.018	0.155	0.506
*DIO1*	0.971	1.451	1.269	1.292	0.208	0.464
(**b**)						
	Treatments *				**SEM**	***p*-Value**
	**Basal**	**0.15 SeMP**	**0.3 SeMP**	**0.6 SeMP/Sel**		
Appetite regulators						
*PYY*	1.093	1.314	0.716	1.146	0.243	0.359
*GLP1*	0.947	1.167	0.882	1.124	0.158	0.519
*NPY*	0.836	0.988	0.915	1.547	0.255	0.199
*CCK*	1.315	1.138	1.203	1.378	0.212	0.849
Tight junctions						
*CLDN1*	0.895	1.144	1.296	0.947	0.208	0.510
*CLDN3*	1.112	1.382	0.898	1.146	0.168	0.251
Nutrient transporters						
*SLC2A2/GLUT2*	1.196	1.112	1.069	1.234	0.180	0.909
*SLC15A1/PEPT1*	1.131	1.189	1.125	1.226	0.166	0.967
*SLC5A1/SGLT1*	0.902 ^a^	1.329 ^b^	0.901 ^a^	1.276 ^b^	0.107	0.037
*FABP2*	1.215	1.271	1.127	1.264	0.167	0.920
Cytokines						
*IFNG*	0.777 ^a^	1.554 ^b^	1.149 ^a,b^	1.212 ^a,b^	0.166	0.027
*TNF*	0.987	1.088	1.034	0.949	0.090	0.720
*TLR4*	0.987	1.048	1.162	1.231	0.165	0.729
*IL10*	1.103	0.996	1.101	1.136	0.171	0.941
*IL6*	0.891	1.012	1.154	1.021	0.248	0.908
*IL17*	0.722	1.531	0.785	1.575	0.275	0.059
*CXCL8/IL8*	1.012	1.130	0.901	1.294	0.170	0.405
Mucins						
*MUC1*	1.254	1.570	0.558	1.080	0.363	0.232
*MUC2*	1.334	1.151	0.699	1.251	0.201	0.138
Selenium transporters						
*SELENOP*	0.664 ^a^	1.058 ^b^	1.252 ^a,b^	1.423 ^c^	0.090	<0.001
*TXNRD1*	0.967	1.054	1.130	1.142	0.134	0.791
*DIO1*	0.830	1.171	0.945	1.173	0.299	0.824
(**c**)						
	Treatments *				**SEM**	***p*-Value**
	**Basal**	**0.15 SeMP**	**0.3 SeMP**	**0.6 SeMP/Sel**		
Appetite regulators						
*PYY*	0.924	1.297	0.865	1.441	0.275	0.382
*NPY*	0.798	1.269	1.054	1.092	0.242	0.625
*CCK*	0.719	0.795	0.913	0.634	0.101	0.211
Tight junctions						
*CLDN1*	1.214	1.340	0.863	1.015	0.200	0.307
*CLDN3*	1.893	1.185	0.642	1.017	0.293	0.059
Nutrient transporters						
*SLC2A2/GLUT2*	1.717	0.880	0.960	1.084	0.270	0.227
*SLC2A5/GLUT5*	1.631	0.866	0.782	1.119	0.246	0.145
*SLC15A1/PEPT1*	1.831	0.896	0.995	1.151	0.256	0.098
*SLC5A1/SGLT1*	1.580	0.999	1.122	1.413	0.276	0.456
*FABP2*	1.578	0.949	1.144	1.572	0.360	0.500
Cytokines						
*IFNG*	1.030	1.054	1.058	1.051	0.152	0.999
*TNF*	0.882	1.302	1.158	0.972	0.158	0.256
*TLR4*	1.299	1.055	1.458	1.082	0.288	0.694
*IL10*	1.126	1.151	1.283	0.983	0.209	0.781
*IL6*	0.769	1.251	1.358	1.150	0.186	0.208
*IL17*	0.997	1.580	0.810	1.953	0.339	0.082
*CXCL8/IL8*	1.297	0.980	1.069	1.169	0.200	0.725
Mucins						
*MUC1*	1.852 ^b^	0.910 ^a^	0.606 ^a^	1.541 ^b^	0.263	0.011
*MUC2*	1.213	1.378	0.668	1.360	0.232	0.098
Selenium tranporters						
*SELENOP*	1.191	0.961	1.125	1.318	0.256	0.781
*TXNRD1*	1.028	1.073	0.902	1.204	0.143	0.505
*DIO1*	0.351	1.150	0.793	0.957	0.205	0.112

Abbreviations: 0.15 SeMP, 0.15 ppm Se (selenium-enriched mushrooms); 0.3 SeMP, 0.3 ppm Se (selenium-enriched mushrooms); 0.6 SeMP/Sel, 0.6 ppm Se (0.3 ppm selenium-enriched mushrooms + 0.3 ppm selenite); *PYY,* peptide YY; *GLP1*, glucagon-like peptide-1; *NPY,* neuropeptide Y; *CCK*, cholecystokinin; *CLND1*, claudin 1; *CLDN3*, claudin 3; *SLC2A2/GLUT2*, glucose transporter 2; *SLC2A5/GLUT5*, glucose transporter 5; *SLC15A1/PEPT1*, peptide transporter 1; *FABP2*, fatty acid binding protein 2; *IFNG*, interferon gamma; *TNF*, tumor necrosis factor alpha; *TLR4*, toll-like receptor 4; *IL10*, interleukin 10; *IL6*, interleukin 6; *IL17*, interleukin 17; *CXCL8*, interleukin 8; *MUC1*, mucin 1; *MUC2*, mucin 2; *SELENOP*, selenoprotein P; *TXNRD1*, thioredoxin reductase 1; *DIO1*; deiodinase 1. ^a,b,c^ Mean values within a row with unlike superscript letters were significantly different (*p* < 0.05). * A total of eight replicates were used per experimental group (replicate = pig). (Least-square mean values with their standard errors).

**Table 9:** Effect of dietary treatment on antioxidant activity, including FRAP and DPPH in the muscle and liver on day 39 post-weaning.

	Treatments *				SEM	*p*-Value
	Basal	0.15 SeMP	0.3 SeMP	0.6 SeMP/Sel		
Muscle						
FRAP	52.39	57.56	51.64	43.94	5.068	0.316
DPPH	15.92	16.48	14.51	14.48	0.813	0.221
Liver						
FRAP	55.12	53.44	49.36	57.483	3.653	0.467
DPPH	15.24	13.43	14.97	14.75	0.641	0.223

Abbreviations: 0.15 SeMP, 0.15 ppm Se (selenium-enriched mushrooms); 0.3 SeMP, 0.3 ppm Se (selenium-enriched mushrooms); 0.6 SeMP/Sel, 0.6 ppm Se (0.3 ppm selenium-enriched mushrooms + 0.3 ppm selenite); FRAP, ferric reducing antioxidant power; DPPH, 2,2-diphenyl-1-picrylhydrazyl. * A total of eight replicates were used per experimental group (replicate = pig). (Least-square mean values with their standard errors).

**Table 10:** Effect of dietary treatment on total selenium content in the muscle and liver on day 39 post-weaning.

	Treatments *				SEM	*p*-Value
	Basal	0.15 SeMP	0.3 SeMP	0.6 SeMP/Sel		
Muscle (µg/kg)	632.78 ^a^	616.59 ^a^	754.88 ^b^	841.92 ^c^	25.009	<0.001
Liver (µg/kg)	1791.69 ^b^	1430.26 ^a^	1685.18 ^b^	2166.21 ^c^	70.569	<0.001

Abbreviations: 0.15 SeMP, 0.15 ppm Se (selenium-enriched mushrooms); 0.3 SeMP, 0.3 ppm Se (selenium-enriched mushrooms); 0.6 SeMP/Sel, 0.6 ppm Se (0.3 ppm selenium-enriched mushrooms + 0.3 ppm selenite) ^a,b,c^ Mean values within a row with unlike superscript letters were significantly different (*p* < 0.05). * A total of eight replicates were used per experimental group (replicate = pig). (Least-square mean values with their standard errors).

## Data Availability

The data presented in this study are available on request from the corresponding author.
